# Adsorption Properties of Low-Cost Biomaterial Derived from *Prunus amygdalus* L. for Dye Removal from Water

**DOI:** 10.1155/2013/961671

**Published:** 2013-07-09

**Authors:** Fatih Deniz

**Affiliations:** Nigar Erturk Trade Vocational High School, 27590 Gaziantep, Turkey

## Abstract

The capability of *Prunus amygdalus* L. (almond) shell for dye removal from aqueous solutions was investigated and methyl orange was used as a model compound. The effects of operational parameters including pH, ionic strength, adsorbent concentration and mesh size, dye concentration, contact time, and temperature on the removal of dye were evaluated. The adsorption kinetics conformed to the pseudo-second-order kinetic model. The equilibrium data pointed out excellent fit to the Langmuir isotherm model with maximum monolayer adsorption capacity of 41.34 mg g^−1^ at 293 K. Thermodynamic analysis proved a spontaneous, favorable, and exothermic process. It can be concluded that almond shell might be a potential low-cost adsorbent for methyl orange removal from aqueous media.

## 1. Introduction

Water is a precious commodity and only an infinitesimal part of the Earth's water reserves (approximately 0.03%) constitutes the water resource that is available for human activities [1]. During the last few decades, the rapid growth of world population and industrial demand has caused serious water pollution. In particular, the textile industry is becoming one of the largest growing industries in the world. Though the textile industry plays an important role in the world economy as well as in our daily life, the colored textile effluent contributes enormously to water deterioration in addition to huge consumption of water and its treatment is the subject of discussion and regulation in many countries. Considering the quantity and composition of the effluent, the textile wastewater is rated as one of the most polluting among all industrial sectors [2]. 

The textile industry accounts for two-thirds of the total dyestuff market. Dyes are used to color the textile products and about 10–15% of the used dyes get lost in the effluent during the dyeing process because of the low level of dye-fiber fixation [3]. The discharges of dye contaminated wastewater into the aquatic environment impact not only the ecological system but also animals and human being. So, the treatment of dye contaminated aquatic systems and improvement of water quality are one of the important topics in the field of environment technologies [4].

In spite of the availability of many techniques (coagulation, chemical oxidation, electrochemical treatments, membrane technologies, etc.) to remove these pollutants from wastewaters, these methods are not very successful due to some drawbacks. On the other hand, adsorption is a very effective separation technique and now it is noted to be superior to other techniques for the water treatment with regard to cost efficiency, eco-friendly, high efficiency, simplicity of design, ease of operation, and insensitivity to toxic substances. Activated carbon is widely used as an adsorbent, but the adsorption by activated carbon has some restrictions including the cost of the activated carbon, the need for regeneration after exhausting, and the loss of adsorption efficiency after regeneration [5]. Therefore, there is a growing interest to search for alternative materials being relatively cost effective and at the same time having high adsorption efficiency. Herein, the use of natural biomaterials is a promising alternative due to their relative abundance and low commercial value.

*Prunus amygdalus* L. (almond) belonging to Rosaceae family is one of the most popular tree nuts and it ranks number one in the tree nut production [6]. It is commercially cultivated in Iran, Italy, Morocco, Spain, Syria, Tunisia, Turkey, USA, and so forth. Worldwide almond production in 2011 was about 2,005,306 metric tons from a total of 1,577,776 hectares [7]. Almond is typically used as snack food and as ingredient in a variety of processed foods, especially in bakery and confectionery products. The edible almond fruit consists of three distinct parts; the inner kernel or meat, the middle shell portion, and an outer green shell cover or hull. When the fruit is processed to obtain the edible seed, big ligneous shell fragments are separated. These materials remain available as a waste product, for which no important industrial use has been developed, so they are normally incinerated or dumped without control. The processing byproducts account for more than 50% by the dry weight of almond fruit [6]. Burning agricultural residues cause environmental problems such as air pollution and soil erosion and they decrease soil biological activity. Utilizing these residues not only prevents environmental concerns but also could mean farmers second income from the plantation [8]. Up to now, there are only few studies reporting the dye removal potential of almond shell [9, 10]. Therefore, the main objects of this research paper are (i) to investigate the feasibility of almond shell for the removal of methyl orange from aqueous solutions, (ii) to determine the various physicochemical controlling factors affecting adsorption including pH, ionic strength, adsorbent concentration and mesh size, dye concentration, contact time, and temperature, (iii) to state kinetic and thermodynamic parameters for explaining the nature of adsorption process, and (iv) to define the practicality of various isotherm models for the best-fit isotherm equation. These data could be useful for further research and the practical applications of almond shell adsorbent in the dyeing wastewater treatment. 

Methyl orange serves as a model pollutant for the common water-soluble azo dyes being widely used in the textile, printing, paper manufacturing, pharmaceutical, food industries, and also in the research laboratories [11]. Because of the common use of such dyes in the industrial applications, the removal of them from industrial wastewaters is capital with regard to protection of public health, environment, and aquatic life.

## 2. Materials and Methods

### 2.1. Almond Shell

Almond shell used in this study was collected from the farmland after almond harvest in Gaziantep province, Turkey. The collected material was first washed with distilled water to remove soluble impurities. It was then dried in an oven for 24 h at 353 K. The dried biomass was powdered and sieved to obtain different mesh size (no) ranges. It was finally stored in an airtight plastic container to use as adsorbent without any pretreatments for the adsorption works.

### 2.2. Methyl Orange

Methyl orange was supplied by Merck KGaA, Darmstadt, Germany. The dye and other reagents were of analytical grade and used without further purification. Properties of methyl orange are presented in Table 1. A stock solution of 500 mg L^−1^ was prepared by dissolving accurately weighed quantity of the dye in distilled water. The working solutions of desired concentrations were then obtained by diluting the dye stock solution with distilled water. The initial pH of solutions was adjusted using 0.1 M HCl and 0.1 M NaOH solutions.

### 2.3. Experimental Design

Adsorption experiments were conducted in batch mode to analyze the effects of various process parameters including pH (3–9), ionic strength (NaCl, 0–0.5 mol L^−1^), adsorbent concentration (1–7 g L^−1^) and mesh size (no) (230–35), dye concentration (50–100 mg L^−1^), contact time (1–140 min), and temperature (293–313 K) under the aspects of isotherms, thermodynamics, kinetics, and mechanism studies. The tests were performed in 100 mL Erlenmeyer flasks with 50 mL of the total working volume of known dye concentration, pH, adsorbent dose, and so forth. The solutions were agitated at a constant speed in a temperature-controlled water bath at different temperatures for the required time period. The flasks were withdrawn from the shaker at predetermined time intervals and the residual dye concentration in the solution was analyzed by centrifuging the mixture and then measuring the absorbance of supernatant using a UV-Vis spectrophotometer at the maximum wavelength of 464 nm. The concentration of methyl orange was calculated by comparing absorbance to the dye calibration curve previously obtained.

The amount of dye adsorbed onto adsorbent (*q*, mg g^−1^) and the percentage dye removal efficiency (*R*, %) were calculated by (1) and (2), respectively,

(1) $$\begin{array}{c} q=\frac{\left(C_{o}-C_{r}\right)V}{M}, \end{array}$$

(2) $$\begin{array}{c} R\left(\%\right)=\frac{C_{o}-C_{r}}{C_{o}}\times 100, \end{array}$$

where *C*_*o*_ is the initial dye concentration (mg L^−1^), *C*_*r*_ is the residual dye concentration at any time (mg L^−1^), *V* is the volume of solution (L), and *M* is the mass of adsorbent (g). *q* and *C*_*r*_ are equal to *q*_*e*_ and *C*_*e*_ at equilibrium, respectively.

### 2.4. Statistical Analysis

All the experiments were performed in duplicates for ensuring the reliability and reproducibility of results obtained and the data were reported as the mean ± SD. The model parameters and constants were analyzed by linear regression using Excel 2010 program (Microsoft Co., USA). In addition to the coefficient of determination (*R* ^2^), the Chi-square (*χ* ^2^) and the mean square error (MSE) test methods were used to evaluate the best-fit of the model to the experimental data using (3) and (4), respectively,

(3) $$\begin{array}{c} \chi^{2}=\sum_{i=1}^{n}\frac{{\left(q_{e,\exp}-q_{e,\text{cal}}\right)}^{2}}{q_{e,\text{cal}}}, \end{array}$$

(4) $$\begin{array}{c} \text{MSE}=\frac{1}{n}\sum_{i=1}^{n}{\left(q_{e,\text{cal}}-q_{e,\exp}\right)}^{2}, \end{array}$$

where *n* is the number of data points, *q*_*e*,exp⁡_ is the observation from the experiment, and *q*_*e*,cal_ is the calculation from the models. The smaller function values point out the best curve fitting.

## 3. Results and Discussion

### 3.1. Effect of pH and Ionic Strength

**Figure 1:**
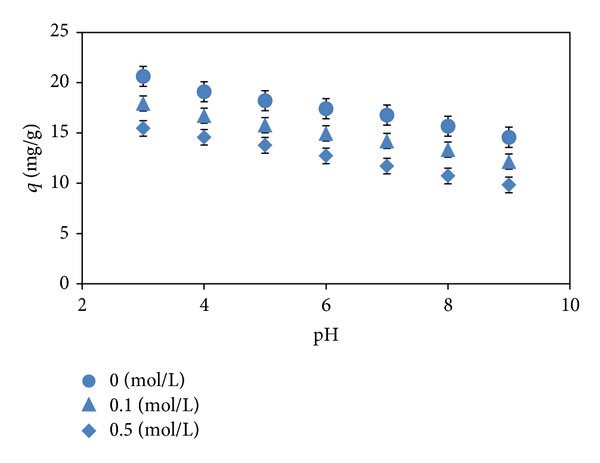
Effect of pH and ionic strength (*m*: 1 g L^−1^, *C*_o_: 50 mg L^−1^, *T*: 293 K).

The pH factor has been recognized as one of the most important impact parameters for dye adsorption process due to influencing the surface property of adsorbent and the ionization degree and speciation of dye molecule. Thus, Figure 1 shows the adsorption profile of methyl orange by almond shell over a pH range of 3–9. The dye removal was found to decrease clearly from 20.63 to 14.57 mg g^−1^ with an increase in pH from 3 to 9. The higher adsorption at very acidic media could be attributed to the electrostatic interactions between the positively charged adsorbent and the negatively charged dye anions. On the contrary, at higher pH values, the number of negatively charged sites on adsorbent increased, which reduced the dye adsorption due to the electrostatic repulsion and also the competition between hydroxyl ions and dye anions for the adsorption sites [4].

Figure 1 also presents the influence of the ionic strength on the adsorption of dye. The occurrence of various types of salts is rather common in colored textile effluents. The salts could change the ionic nature, hydrophobicity, size, and solubility of the dye and their presence leads to high ionic strength, which may significantly affect the performance of dye adsorption process [12]. Thus, besides pH, it is useful to discuss the effect of salt concentrations on the adsorption behavior of adsorbent. In this work, sodium chloride (0–0.5 mol L^−1^) was used to simulate the salt ionic strength in colored wastewaters. Although the ionic strength did not affect the pH trend of adsorption, it influenced the dye removal adversely. Thus, increased ionic strength led to a decrease in the dye adsorption potential of adsorbent. This phenomenon may be explained by the possibility of ion exchange mechanism or the competition between chloride anions and negatively charged dye molecules for the same binding sites.

### 3.2. Effect of Adsorbent Concentration and Mesh Size

**Figure 2:**
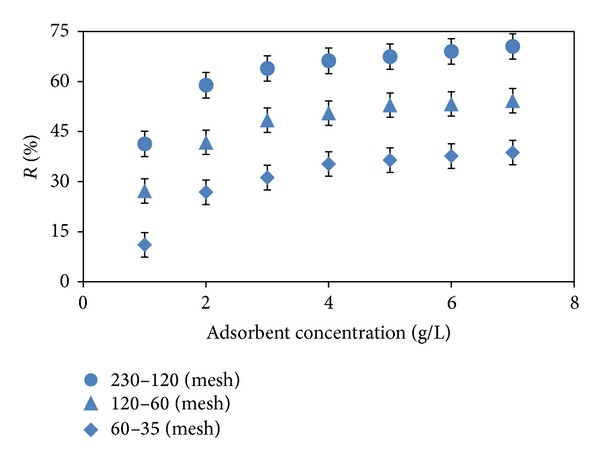
Effect of adsorbent concentration and mesh size (pH: 3, *C*_*o*_: 50 mg L^−1^, *T*: 293 K).

The adsorbent dose is an important parameter in the adsorption studies because it gives an idea of the adsorbent efficiency.  Figure 2 represents the adsorption yield of methyl orange versus almond shell concentration in the range of 1–7 g L^−1^. It was observed that percentage of dye removal increased with increase of adsorbent dose. Such a trend is mostly attributed to an increase in the adsorptive surface area and the availability of more active adsorption sites [13].

Figure 2 also shows the effect of mesh size at different levels ranging from 230 to 35 (63–500 *µ*m) on the dye adsorption. The results indicated that the dye removal enhanced with decreasing the adsorbent particle size. The higher dye removal efficiency with smaller particles can be due to the fact that smaller adsorbent particles provide a larger surface area and better accessibility of dye into active pores [14].

### 3.3. Effect of Dye Concentration

The dye concentration has an apparent influence on its removal from aqueous phase. The effect of methyl orange concentration on the efficiency of adsorption was also investigated in the initial concentration range of 50–100 mg L^−1^ (figure not shown). The adsorption capacity of almond shell at equilibrium increased from 23.42 to 40.21 mg g^−1^ with increase in the initial dye concentration from 50 to 100 mg L^−1^ (*m*: 1 g L^−1^, pH: 3, *T*: 293 K). This trend may be due to the high driving force for mass transfer at a high initial dye concentration. In addition, if the dye concentration in solution is higher, the active sites of adsorbent are surrounded by much more dye molecules and the adsorption phenomenon occurs more efficiently. Thus, adsorption amount increases with the increase of initial dye concentration [14].

### 3.4. Effect of Temperature and Contact Time

**Figure 3:**
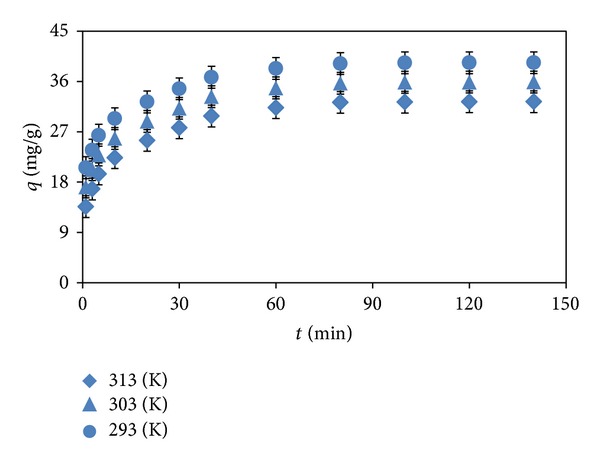
Effect of temperature and contact time (pH: 3, *m*: 1 g L^−1^, *C*_o_: 100 mg L^−1^).

Temperature is a significant controlling factor in the real applications of adsorbent for the dye removal process.  Figure 3 presents the adsorption of methyl orange by almond shell at different temperatures as a function of contact time. The temperature negatively affected the dye adsorption efficiency. The adsorption capacity of adsorbent at equilibrium decreased from 40.21 to 32.37 mg g^−1^ with an increase in temperature from 293 to 313 K. This decrease may be due to weakening of the bonds between the dye molecules and the active sites of adsorbent [4].

Also, the dye adsorption was rapid in the initial stages of removal process and increased with an increase in contact time up to 80 min. After this period, the adsorption amount did not significantly change up to 140 min. The fast initial adsorption rate may be attributed to a large number of the vacant dye binding sites being available for adsorption during the initial stage. At higher contact time, the rate of adsorption inclined to slow down, gradually leading to equilibrium. This trend could be referred to the decrease in the number of vacant sites being available for further dye removal [14]. The short equilibrium time points out the efficiency and applicability of adsorbent for real wastewater treatment process.

### 3.5. Kinetic Characteristics

The adsorption kinetic parameters are useful for the prediction of adsorption rate giving considerable information for designing and modeling adsorption process, operation control, and adsorbent evaluation. Thus, the pseudo-first-order [19] and pseudo-second-order [20] rate equations were used to study the adsorption kinetics by (5) and (6), respectively,

(5) $$\begin{array}{c} \frac{1}{q_{t}}=\frac{1}{q_{e}}+\frac{k_{1}}{q_{e}t}, \end{array}$$

(6) $$\begin{array}{c} \frac{t}{q_{t}}=\frac{1}{k_{2}q_{e}^{2}}+\frac{t}{q_{e}}. \end{array}$$

**Figure 4:**
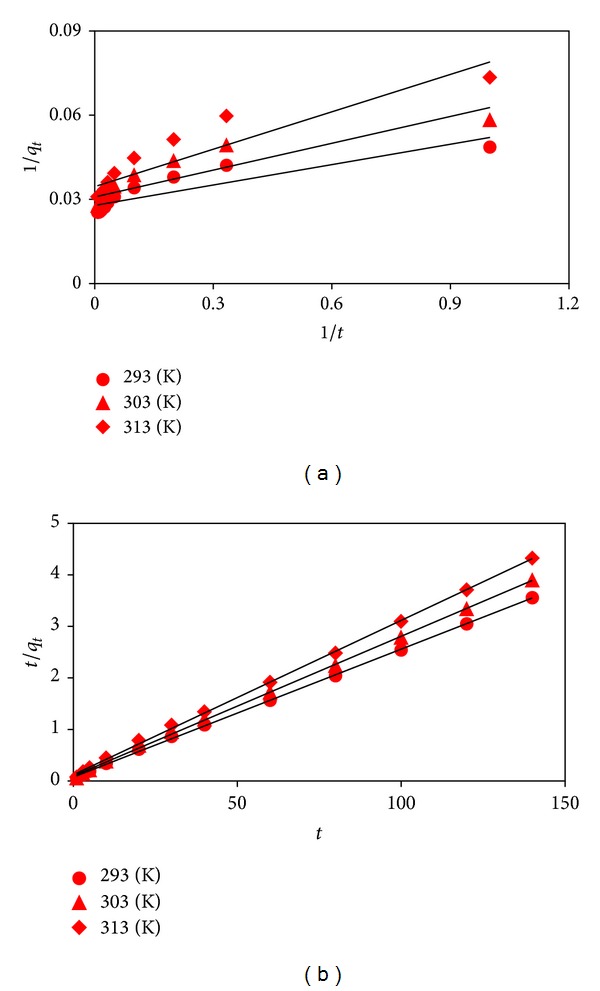
Plots for pseudo-first-order (a) and pseudo-second-order (b) kinetic models.

Also, the initial adsorption rate, *h* (mg g^−1^ min^−1^), is determined by (7)

(7) $$\begin{array}{c} h=k_{2}q_{e}^{2}, \end{array}$$

where *k*_1_ is the pseudo-first-order rate constant (min^−1^), *k*_2_ (g mg^−1^ min^−1^) is the constant of pseudo-second-order rate for the adsorption, and *q*_*e*_ and *q*_*t*_ (mg g^−1^) are the amounts of dye adsorbed at equilibrium and at time *t*, respectively. The values of *k*_1_ and *q*_*e*_ can be calculated from the intercept and slope of the plots of 1/*q*_*t*_ versus 1/*t* (Figure 4(a)) for the pseudo-first-order model while *k*_2_ and *q*_*e*_ values can be obtained from the slope and intercept of the plots of *t*/*q*_*t*_ versus *t* (Figure 4(b)) for the pseudo-second-order model. All the calculated model parameters and constants with the statistical analysis values are presented in Table 2. The low *R* ^2^ beside high *χ* ^2^ and MSE values for the pseudo-first-order model show that the model was not favorable for defining the adsorption kinetics. Contrary to the pseudo-first-order model, the relatively high *R* ^2^ as well as small *χ* ^2^ and MSE values for the pseudo-second-order model suggest that the adsorption process obeyed the model kinetics for all temperatures (293–313 K). This attitude proves that the rate-limiting step is probably the surface adsorption for the dye adsorption process [14]. Additionally, the values of *k*_2_ and *h* decreased with increase in temperature suggesting that they were affected by the temperature.

### 3.6. Intraparticle Diffusion and Adsorption Mechanism

**Figure 5:**
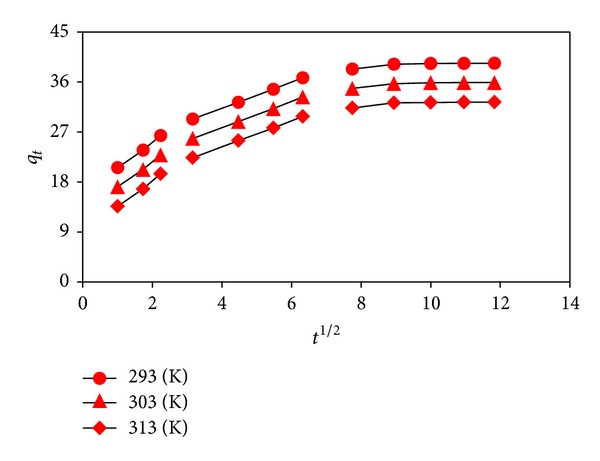
Intraparticle diffusion model plots.

Since the pseudo-first-order and pseudo-second-order kinetic models do not identify the adsorption diffusion mechanism, the intraparticle diffusion model [21] was further used to define the rate-controlling step(s) by the following equation:

(8) $$\begin{array}{c} q_{t}=k_{p}t^{1/2}+C, \end{array}$$

where *k*_*p*_ is the intra-particle diffusion rate constant (mg g^−1^ min^−1/2^) and *C* (mg g^−1^) is a constant providing information about the thickness of boundary layer, which can be calculated from the intercept and slope of the plots of *q*_*t*_ versus *t* ^1/2^. According to the model, if the plot of *q*_*t*_ versus *t* ^1/2^ gives a straight line passing through the origin, then the adsorption process is controlled by the intra-particle diffusion, while if the data exhibit multilinear plots, then two or more steps influence the process. The plots for the dye adsorption by almond shell at different temperatures were multimodal with three distinct regions (Figure 5). The initial curved region corresponds to the external surface adsorption, in which the dye diffuses through the solution to the external surface of adsorbent. The second stage relates the gradual adsorption reflecting intra-particle diffusion as the rate-controlling step. The final plateau region points out the surface adsorption and the equilibrium stage, in which the intra-particle diffusion starts to slow down and level out [13, 14]. Based on the present results (Figure 5 and Table 2), it could be inferred that the intra-particle diffusion was involved in the adsorption process, but it was not the only rate-limiting step and that the other step(s) along with intra-particle diffusion might be also involved.

### 3.7. Equilibrium Isotherms

The adsorption isotherms provide some insight into the adsorption mechanism and the affinity and surface characteristics of adsorbent. So in this research, the Freundlich [22] and Langmuir [23] isotherm models were used to define the equilibrium data by (9) and (10), respectively,

(9) $$\begin{array}{c} \ln q_{e}=\ln K_{f}+\frac{1}{n_{f}}\ln C_{e}, \end{array}$$

(10) $$\begin{array}{c} \frac{C_{e}}{q_{e}}=\frac{1}{bq_{m}}+\frac{C_{e}}{q_{m}}. \end{array}$$

**Figure 6:**
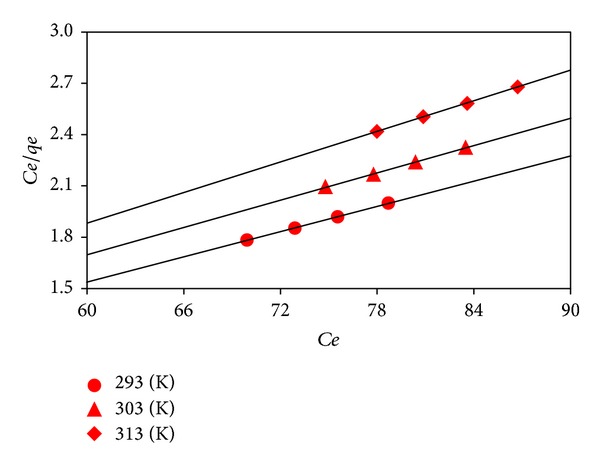
Langmuir isotherm model plots.

Else, the separation factor, *R*_*L*_ (the essential point of Langmuir model), is specified by the following equation:

(11) $$\begin{array}{c} R_{L}=\frac{1}{1+bC_{o}}, \end{array}$$

where *K*_*f*_ (mg g^−1^) (L g^−1^)^1/*n*^ is the constant related to adsorption capacity, *n*_*f*_ is the parameter related to the adsorption intensity, *b* (L mg^−1^) is the constant related to the energy of adsorption, and *q*_*m*_ is the maximum monolayer adsorption capacity (mg g^−1^). *K*_*f*_ and *n*_*f*_ values can be determined from the slope and intercept of the plots between ln *q*_*e*_ and ln *C*_*e*_ (plots not shown) for the Freundlich model while the values of *b* and *q*_*m*_ can be calculated from the slope and intercept of the plots between *C*_*e*_/*q*_*e*_ and *C*_*e*_ (Figure 6) for the Langmuir model. For adsorption isotherms, all the obtained model parameters and constants along with the statistical data at different temperatures are listed in Table 3. The low *R* ^2^ as well as high *χ* ^2^ and MSE values for the Freundlich model prove that this model was not practicable for describing the equilibrium isotherms. Unlike the Freundlich model, the comparatively high *R* ^2^ beside small *χ* ^2^ and MSE values for the Langmuir model propose that the adsorption process took place at the specific homogeneous sites within the adsorbent surface and that once the dye molecule occupied a site, no further adsorption could take place at that site, which concluded that the adsorption process was monolayer in nature [24]. In addition, the values of *K*_*f*_, *q*_*m*_, and *b* decreased with the increase in temperature. The bigness of *n*_*f*_ gives a measure of the suitability of adsorption. The value of *n*_*f*_ between 1 and 10 points a favorable adsorption [13]. For this work, the values of *n*_*f*_ showed the same trend presenting a profitable adsorption. The *R*_*L*_ value between 0 and 1 also reflects an agreeable adsorption [25]. The *R*_*L*_ values for the dye removal were obtained at the range of 0.027–0.038 denoting that the adsorption was an applicable process.

Table 4 outlines the comparison of *q*_*m*_ value of various adsorbents including almond shell for the methyl orange adsorption. Almond shell has higher adsorption capacity of the dye in comparison with many of the other reported adsorbents. Thus, it could be used as a promising clean-up material for the dye contaminated wastewaters.

### 3.8. Thermodynamic Parameters and Activation Energy

The thermodynamic data including the standard Gibbs free energy change (Δ*G*°, kJ mol^−1^), standard enthalpy change (Δ*H*°, kJ mol^−1^), and standard entropy change (Δ*S*°, kJ mol^−1^ K^−1^) have an important role to state the feasibility, spontaneity, and nature of adsorption process. These parameters can be calculated by (12) [10]:

(12) $$\begin{array}{c} {\Delta G}^{{}^{\circ}}=-RT\ln K_{c},\\ \ln K_{c}=\frac{{\Delta S}^{{}^{\circ}}}{R}-\frac{{\Delta H}^{{}^{\circ}}}{RT}, \end{array}$$

where *R* is the universal gas constant (8.314 J mol^−1^ K^−1^), *T* is the temperature (K), *K*_*c*_ is the distribution coefficient (*C*_*s*_/*C*_*e*_), *C*_*s*_ and *C*_*e*_ are the equilibrium dye concentrations on adsorbent (mg L^−1^) and in solution (mg L^−1^), respectively. Δ*S*° and Δ*H*° values can be obtained from the slope and intercept of the plot of ln *K*_*c*_ versus 1/*T* (plot not shown). All the determined thermodynamic parameters are given in Table 5. The negative values of Δ*G*° at all studied temperatures indicated the feasibility and spontaneous nature of dye adsorption process. Likewise, decrease in Δ*G*° value with increasing temperature suggests that the adsorption was more favorable at lower temperatures. The negative value of Δ*S*° reflects that the dye removal process was enthalpy driven while the negative value of Δ*H*° implies that the adsorption reaction was exothermic [13].

The Arrhenius equation was further used to calculate the activation energy (*E*_*a*_, kJ mol^−1^) for the dye adsorption by (13) [19]

(13) $$\begin{array}{c} \ln k_{2}=\ln A-\frac{E_{a}}{RT}, \end{array}$$

where *k*_2_ is the constant of the pseudo-second-order rate (g mg^−1^ min^−1^) and *A* is the Arrhenius constant. The value of *E*_*a*_ can be determined from the intercept of the plot of ln⁡*k*_2_ versus 1/*T* (plot not shown). The magnitude of *E*_*a*_ gives an idea about the type of adsorption (physical or chemical). According to the literature, the methyl orange adsorption by almond shell may be physical adsorption [14].

## 4. Conclusions

In this work, adsorption potential of almond shell to remove methyl orange as a model pollutant from aqueous solutions was investigated in batch system. The pH, ionic strength, adsorbent concentration and mesh size, dye concentration, contact time, and temperature played a significant role in the dye removal capacity of adsorbent. The adsorption process obeyed the pseudo-second-order kinetics well. The Langmuir isotherm model presented the best fit to experimental data. Thermodynamic parameters showed the exothermic and spontaneous nature of dye adsorption. Consequently, almond shell could be used as a promising clean-up material for methyl orange contaminated wastewaters.

## Figures and Tables

**Table 1:** Properties of methyl orange.

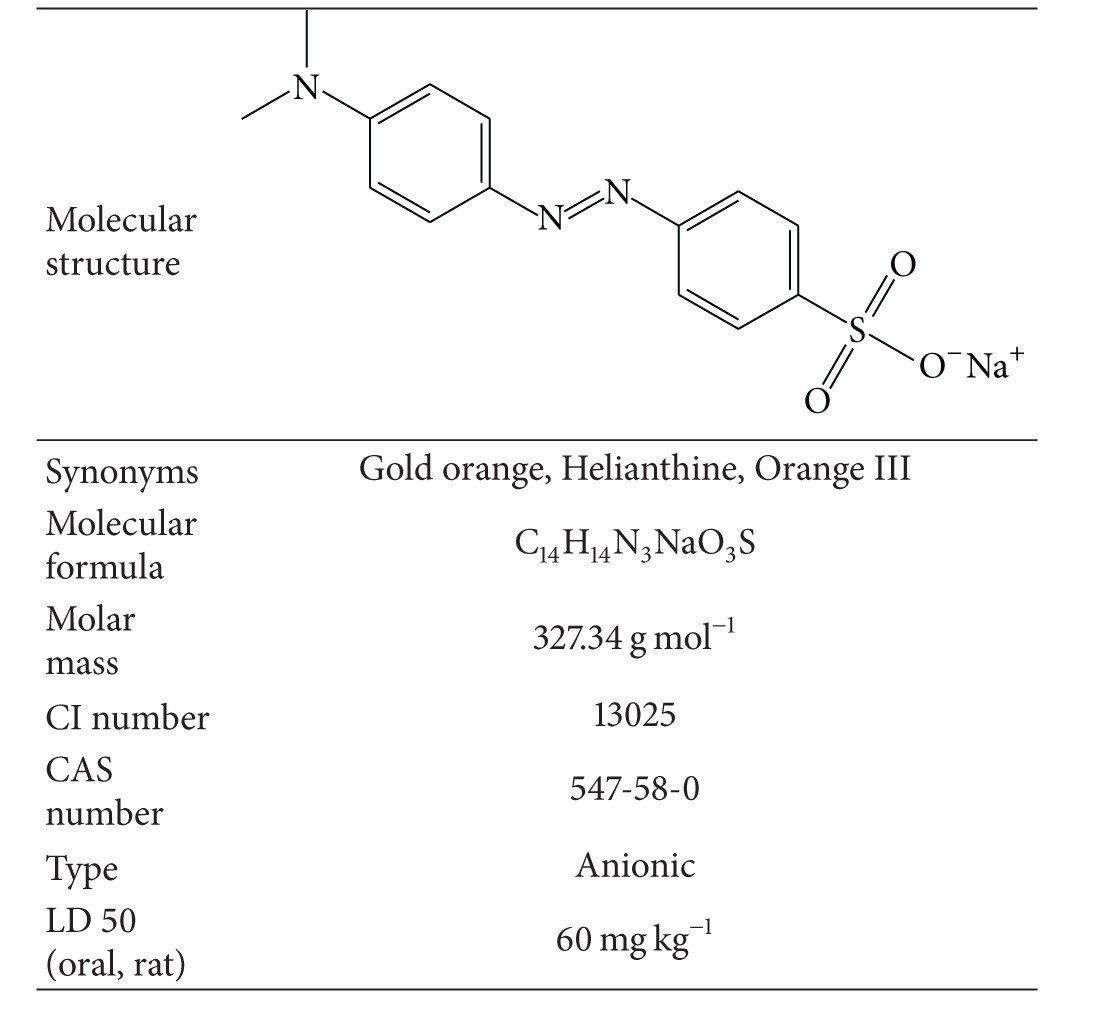

**Table 2:** Kinetic model parameters and constants with statistical data.

Model	Parameter	Temperature (K)		
		293	303	313
Pseudo-first-order	*q*_*e*,cal_ (mg g^−1^)	34.75	32.47	28.98
	*k*_1_ (min^−1^)	0.915	1.032	1.287
	*R* ^2^	0.8038	0.8173	0.8470
	χ^2^	2.839	3.221	3.413
	MSE	10.163	9.887	9.307

Pseudo-second-order	*q*_*e*,cal_ (mg g^−1^)	39.57	36.90	33.44
	*k*_2_ (g mg^−1^ min^−1^)	0.00811	0.00767	0.00735
	*h* (mg g^−1^ min^−1^)	12.70	10.44	8.22
	*R* ^2^	0.9997	0.9993	0.9992
	χ^2^	0.455	0.459	0.460
	MSE	1.494	1.362	1.213

Intraparticle diffusion	*C* (mg g^−1^)	21.89	19.43	15.99
	*k*_*p*_ (mg g^−1^ min^−1/2^)	1.6912	1.6831	1.6769
	*R* ^2^	0.8814	0.8703	0.8680
	χ^2^	1.193	1.382	1.558
	MSE	4.465	4.479	4.496

	*q*_*e*,exp⁡_ (mg g^−1^)	40.21	35.89	32.37

**Table 3:** Parameters of isotherm models with statistical analysis values.

Model	Parameter	Temperature (K)		
		293	303	313
Freundlich	*K*_*f*_ (mg g^−1^) (L g^−1^)^1/*n*^	11.025	7.354	5.305
	*n*_*f*_	3.167	2.765	2.445
	*R* ^2^	0.7016	0.7136	0.9065
	*χ* ^2^	5.378	5.145	3.947
	MSE	13.435	13.132	7.638

Langmuir	*b* (L mg^−1^)	0.354	0.306	0.254
	*q*_*m*_ (mg g^−1^)	41.34	37.59	33.56
	*R*_*L*_	0.027	0.032	0.038
	*R* ^2^	0.9989	0.9990	0.9998
	*χ* ^2^	0.411	0.402	0.393
	MSE	1.657	1.643	1.421

	*q*_*e*,exp⁡_ (mg g^−1^)	40.21	35.89	32.37

**Table 4:** Comparison of methyl orange adsorption capacity of various adsorbents.

Adsorbent	Conditions	*q*_*m*_ (mg g^−1^)	Reference
Waste beer yeast biomass	pH: 6, *M*: 0.02 g, *V*: 0.04 L, *C*_*o*_: 327.34 mg L^−1^, *T*: 298 K, *t*: 50 min	3.0	[15]

Bottom ash	pH: 3, *M*: 0.1 g, *V*: 0.025 L, *C*_*o*_: 32.734 mg L^−1^, *T*: 303 K, *t*: 240 min	3.6	[16]

Balsam fir wood sawdust	pH: 6, *M*: 0.8 g, *V*: 0.05 L, *C*_*o*_: 50 mg L^−1^, *T*: 25°C, *t*: 60 min	7.7	[17]

Granulated activated carbon	pH: 6, *M*: 0.8 g, *V*: 0.05 L, *C*_*o*_: 50 mg L^−1^, *T*: 298 K, *t*: 60 min	10.3	[17]

Deoiled soya	pH: 3, *M*: 0.05 g, *V*: 0.025 L, *C*_*o*_: 32.734 mg L^−1^, *T*: 303 K, *t*: 150 min	16.7	[16]

Orange peel	pH: 7, *M*: 0.1 g, *V*: 0.1 L, *C*_*o*_: 50 mg L^−1^, *T*: 303 K, *t*: 65 min	20.5	[18]

Banana peel	pH: 6, *M*: 0.1 g, *V*: 0.1 L, *C*_*o*_: 50 mg L^−1^, *T*: 303 K, *t*: 65 min	21.0	[18]

Almond shell	pH: 3, *M*: 0.05 g, *V*: 0.05 L, *C*_*o*_: 100 mg L^−1^, *T*: 293 K, *t*: 140 min	41.34	This study

**Table 5:** Thermodynamic parameters.

Parameter	Temperature (K)		
	293	303	313
−Δ*G*° (kJ mol^−1^)	6.543	6.063	5.687
−Δ*H*° (kJ mol^−1^)	16.869		
−Δ*S*° (kJ mol^−1^ K^−1^)	0.0353		
*E*_*a*_ (kJ mol^−1^)	3.309		
